# Fostamatinib and the risk of acute aortic dissection in immune thrombocytopenia

**DOI:** 10.1002/bcp.70289

**Published:** 2025-09-17

**Authors:** Paul Dalmas, Alexis Theron, Rita Badaoui, Stéphane Zaffran, Rayid Mchinda, Joelle Micallef, Nicolas Schleinitz, Mikael Ebbo

**Affiliations:** ^1^ Aix Marseille University, APHM, Internal Medicine Department La Timone Hospital Marseille France; ^2^ Department of cardiac Surgery La Timone Hospital Marseille France; ^3^ Aix‐Marseille University, INSERM MMG U1251 Marseille France; ^4^ Radiology Department La Timone Hospital, Assistance Publique Des Hôpitaux de Marseille Marseille 05 France; ^5^ Aix Marseille University, APHM, Pharmacologie Clinique & Pharmacosurveillance Department Sainte Marguerite Hospital and Regional Pharmacovigilance Center Marseille France

**Keywords:** adverse drug reactions, cardiology, clinical pharmacology, drug safety, immunology, vascular disease

## Abstract

Immune thrombocytopenia (ITP) is a rare autoimmune disorder characterized by platelet destruction. While most patients respond to first‐ or second‐line therapies, a small subset is multirefractory. Fostamatinib, an oral spleen tyrosine kinase (SYK) inhibitor, is a therapeutic option in these cases. We report a case of an acute aortic dissection (AAD) occurring in a patient without traditional cardiovascular risk factors, 3 months after fostamatinib initiation. A 70‐year‐old woman with a 30‐year history of ITP, unresponsive to multiple therapies, was treated with fostamatinib and achieved a complete haematologic response. She presented with sudden chest pain and was diagnosed with Stanford A (DeBakey type 1) AAD requiring emergency surgery. Histological analysis of her aortic tissue showed a 25% reduction in phosphorylated SYK expression compared to a control sample, without alteration in smooth muscle cell markers. The absence of predisposing conditions (hypertension, smoking, genetic disorders) led us to explore a potential link between fostamatinib and AAD. Experimental data suggest that SYK inhibition exacerbates aortic wall vulnerability, and off‐target effects of fostamatinib on VEGF receptors may further weaken vascular integrity. This case highlights a possible association between fostamatinib and AAD, potentially mediated by SYK and VEGFR inhibition. While causality cannot be definitively established, clinicians should be vigilant when prescribing fostamatinib, even in patients without known risk factors for AAD. Further studies are needed to clarify the vascular safety profile of fostamatinib in ITP and other settings.

## 1 INTRODUCTION

Immune thrombocytopenia (ITP) is a rare disease characterized by immune destruction of platelets.^1^ According to guidelines, first‐line treatments are based on corticosteroids or polyvalent intravenous immunoglobulins, while second‐line therapies include rituximab, thrombopoietin‐receptor agonists or splenectomy.^2^ Between 2 and 3% of patients are considered multirefractory because they do not respond to second‐line treatments^3^ and these situations require other therapies. Notably, fostamatinib, an oral spleen tyrosine kinase (SYK) inhibitor, has demonstrated efficacy in patients refractory to other treatments.^4^ The use of fostamatinib for ITP in clinical practice shows a good safety profile, with the main side effects being diarrhoea and hypertension, observed respectively in 20% and 15% of patients.^5^

Acute aortic dissection (AAD) is a rupture of the aortic wall, either due to an intimal tear or an intramural haematoma.^6^ The Stanford classification distinguishes acute aortic dissections involving the ascending aorta (Stanford type A) from those that do not (Stanford type B). This rare condition requires prompt management, primarily through open surgery or endovascular intervention, both accompanied by medical measures. Classical risk factors for AAD are well identified, such as long‐term arterial hypertension, smoking or connective tissue disorders.^6^ Moreover, new factors have been recently identified, including medications such as antivascular endothelial growth factor receptor (VEGFR) agents such as sorafenib and regorafenib.^7^

Here, we present a case of AAD in a patient without any risk factors, receiving fostamatinib for ITP, and discuss the potential link between fostamatinib and AAD.

## 2 CASE REPORT

A 70‐year‐old woman presented to the emergency department with sudden thoracic pain and transient loss of consciousness without trauma or stress at the time of onset. Her medical history included a diagnosis of ITP 30 years prior. Despite transient responses to oral corticosteroids and intravenous immunoglobulins, she did not respond to splenectomy, romiplostim, eltrombopag and, after initial response, was contraindicated to rituximab due to severe anaphylactic reaction. She did not have any arterial hypertension, aortic aneurysm, bicuspid aortic valve, connective tissue disorders or tobacco use. She did not have family history of AAD, aortic aneurysm or bicuspid aortic valve. Due to recurrent episodes of thrombocytopenia below 20 × 10^9^/L with severe bleeding, she began treatment with oral fostamatinib (100 mg twice daily) 3 months prior with complete response (platelets between 150 and 200 × 10^9^/L) and good tolerance, especially on blood pressure that was monitored daily and was within the normal range.

**FIGURE 1:**
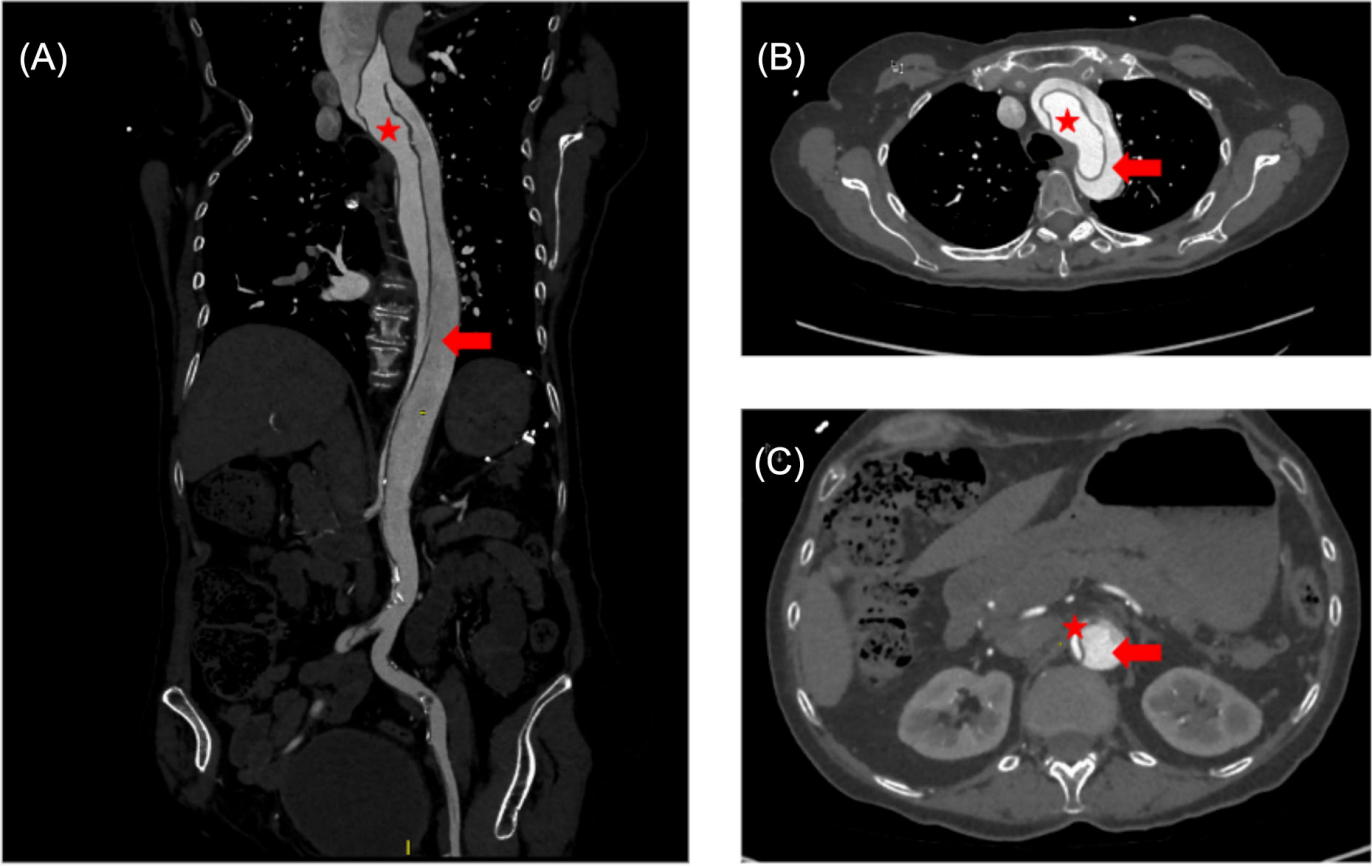
Computed tomography imaging of acute aortic dissection. (A) curvilinear reconstruction of the descending aorta showing AAD involving thoracic aorta, abdominal aorta and iliac artery, (B) thoracic aorta, (C) abdominal aorta. Red stars represent true lumen and red arrows represent false lumen.

Upon examination, she exhibited bradycardia (40 beats per minute), asymmetrical arm blood pressure (difference of 50 mm of Hg between left and right arm) and absent left femoral pulse. Laboratory tests revealed mild thrombocytopenia (128 × 10^9^/L; normal range: 150–400 × 10^9^/L), with normal renal and hepatic functions. Transthoracic echocardiography ruled out any significant aortic regurgitation. A computed tomography scan showed a Stanford A aortic dissection extending from the tubular ascending aorta to the abdominal aorta, corresponding to a DeBakey type 1 dissection, together with a pericardial effusion (Figure 1). She underwent a replacement of the tubular portion of the ascending aorta associated to an open arch anastomosis with a prosthetic Dacron graft under extracorporeal circulation. Surgery was complicated by early postoperative haemodynamic instability, consecutive to cardiac tamponade requiring external drainage, and ischaemic stroke attributed to low flow with subsequent left hemiplegia (strength estimated at 1/5 according to the Medical Research Council scale with muscle contraction visible without movement). After a few days, the patient developed thrombocytopenia with 29 × 10^9^/L of platelets with good haematological response to intravenous methylprednisolone at 100 mg per day for 5 days followed by oral prednisone at 10 mg per day. After 20 days, at discharge, her hemiplegia partially improved but she kept left hemiparesis (strength estimated at 4/5 according to Medical Research Council scale with active movement possible against resistance despite reduced strength), and repeat imaging showed stability of the aortic dissection from the aortic arch to the iliac artery and the platelets were 625 × 10^9^/L. Fostamatinib was definitely stopped, and prednisone was initially continued at 10 mg per day in order to avoid new episodes of thrombocytopenia.

Two months after the AAD, she was successfully treated with obinutuzumab, an anti‐CD20 monoclonal antibody, 1 g Day 1 and 1 g Day 15 and oral corticotherapy was stopped. Four months post‐dissection, imaging revealed stability of the AAD without prosthetic anomalies. At 20 months after treatment with obinutuzumab, she relapsed with thrombocytopenia at 6 × 10^9^/L and gingival bleeding. Due to poor biological and clinical outcome after oral prednisone tapered for 3 weeks and intravenous immunoglobulins, new infusions of obinutuzumab 1 g Day 1 and Day 15 were successfully administered and resulted in complete haematological response. The long‐term management plan for this patient's ITP includes regular monitoring of platelet counts, gradual tapering of prednisone to 5 mg followed by discontinuation, and biannual assessment of B cell levels to determine the need for additional obinutuzumab infusions in the event of relapse.

**FIGURE 2:**
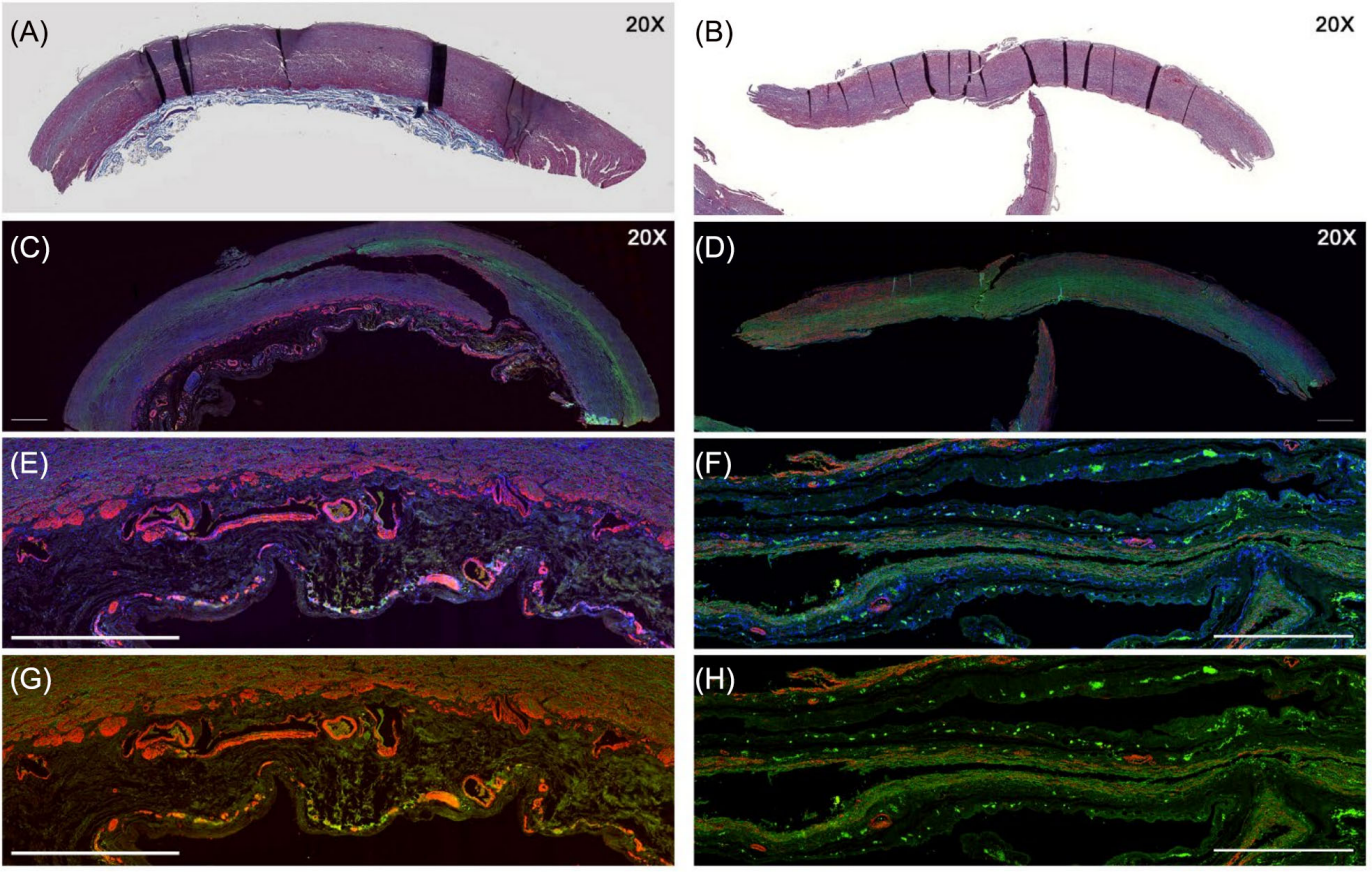
Aortic biopsy. The left panels show histology from the control patient (AAD without fostamatinib), while the right panels display histology from the patient (AAD with fostamatinib). Panels A and B present Masson’ trichrome staining of the aortic biopsy from both the control and the patient (magnification ×20). Panels C, D, E, F, G and H represent immunofluorescence labelling of p‐SYK (red), decreased in patient treated with fostamatinib (panels D, F and H) and αSMA (green), unchanged in patient treated with fostamatinib. The nuclei (blue) were stained with DAPI on panels C, D, E and F. Magnification in panels C and D is ×20. Panels E and G, as well as F and H, show higher numeric magnifications of the aorta depicted in panels C and D, respectively.

As the patient does not have a personal or familial risk factor of AAD, we decided to analyse the patient's aortic biopsy and compare it to another AAD biopsy from a control patient not treated with fostamatinib (Figure 2). We focused on SYK expression and smooth muscle cells (SMCs) in the patient's aortic biopsy using respectively phosphorylated SYK (pSYK) and α smooth muscle actin (αSMA). In the patient's aortic specimen, we observed a decrease of 25% of pSYK expression compared with the control aortic specimen. No difference was observed concerning αSMA. Methods of analysis are available in Supplemental 1.

## 3 DISCUSSION

We present here a case of a patient who developed Stanford A AAD 3 months after fostamatinib initiation for refractory ITP. The occurrence of AAD in this case was unexpected because of the absence of personal risk factors such as arterial hypertension, smoking, connective tissue disorders, aortic aneurysm, bicuspid aortic valve or familial risk factors. The introduction of fostamatinib 3 months prior led us to hypothesize a link between this treatment and AAD. Indeed, Kang et al.^7^ have described an association between the use of tyrosine kinase inhibitor targeting VEGFR and the development of AAD with highest incidence at 3 months following initiation of treatment and among women. Hence to understand this case, we briefly review the pathogenesis of AAD, the mechanisms of action of fostamatinib, and explore the potential link between fostamatinib and AAD.

Mechanistically, AAD occurs when blood infiltrates the media of the aortic wall, typically through an intimal tear. The intima, composed of endothelial cells, forms the inner layer of the aortic wall, while the media, consisting of connective tissue with smooth muscle cells and elastic lamellae, constitutes the middle layer. Broadly, risk factors can contribute to AAD through two mechanisms: heightened aortic wall stress, primarily due to elevated blood pressure, and increased aortic wall weakness, as seen in connective tissue disorders, aortitis^8^ and also with anti‐VEGFR therapy.^7^

SYK is a cytoplasmic protein possessing tyrosine kinase properties that play a pivotal role in the signalling of fragment crystallizable receptor (FcRs) and B cell receptor (BCR).^9^ Fostamatinib undergoes conversion to its active form, R406, within the gastrointestinal tract, and inhibits SYK tyrosine kinase activity by competing with adenosine triphosphate (ATP) binding site.^9^ This inhibition of SYK disrupts FcRs signalling, reducing the phagocytosis of opsonized platelets by splenic macrophages. Additionally, it interferes with BCR function, resulting in reduced production of antiplatelet antibodies by B cells.^9^ However, fostamatinib's action is not exclusive to SYK and inhibits other kinases, such as VEGFR, in an off‐target manner, leading to the development of arterial hypertension.^10^

Hashimoto et al. demonstrated that SYK is activated in a mouse model of AAD, and inhibition with fostamatinib exacerbated the length of aortic lesions and mortality.^11^ Specifically, fostamatinib suppresses phospho‐SYK and the signal transducer and activator of transcription 3 (STAT‐3), which is known to provide protection against AAD.^11^ Additionally, SYK activation has been observed in macrophages and SMCs in AAD.^12^ However, the finding that models with SYK knocked out in macrophages and SMCs do not show a worse prognosis suggests that the effects of fostamatinib on SYK may involve other cell types as well.^12^

As noted earlier, fostamatinib can also inhibit VEGFR, and the link between AAD and anti‐VEGFR therapy is well documented, with multifactorial underlying causes. One potential explanation is that VEGFR inhibition induces systemic vasoconstriction and volume overload, both of which increase arterial blood pressure and exacerbate aortic wall tension.^13^ However, in this case, this hypothesis seems less plausible as the patient's arterial blood pressure was well‐controlled whereas AAD is typically linked to long‐standing arterial hypertension.^6^

Another potential explanation is that VEGF inhibition compromises the integrity of the aortic wall. Firstly, it leads to the inhibition of phosphatidylinositol‐3‐kinase‐Akt, resulting in the overexpression of matrix metalloproteinase 9 (MMP9), which degrades the extracellular matrix.^7^ Additionally, VEGF inhibition is responsible for the apoptosis of SMCs^14^ and impaired endothelial regeneration.^7^

Similar to the findings in the previous mouse model, the histological study of our patient (Figure 2) exhibits reduction of pSYK expression in the aortic wall suggesting a potential deleterious effect and a role for fostamatinib in the development of AAD.^11^, ^12^ However, no difference was observed for αSMA, which reflects the number of SMCs, suggesting that there was no increase in SMC apoptosis in our case.

In conclusion, we present the first reported case of AAD in a patient with refractory ITP managed with fostamatinib. We propose that this treatment may have predisposed the patient to AAD through SYK inhibition and off‐target inhibition of VEGFR, which weakened the aortic wall. We emphasize the need for careful consideration when using fostamatinib in patients with risk factors for AAD, even if this event appears to be possible in patients without risk factors, as in our case.

### 3.1 Nomenclature of targets and ligands

Key protein targets and ligands (SYK, VEGFR, fostamatinib, obinutuzumab, sorafenib and regorafenib in this article have been hyperlinked to corresponding entries in http://www.guidetopharmacology.org, and are permanently archived in the Concise Guide to PHARMACOLOGY 2023/24.^15^, ^16^, ^17^

## AUTHOR CONTRIBUTIONS

P.D. and M.E. wrote the manuscript. M.E. supervised the study. A.T., R.B., R.M., J.M., S.Z. and N.S. critically reviewed the manuscript.

## CONFLICT OF INTEREST STATEMENT

All authors declare there are no conflicts of interest.

## Supporting information


**Data S1.** Histological and Immunostaining.

## Data Availability

Original data and protocol on analysis are available by contacting the corresponding author Paul Dalmas. The authors declare that they have obtained written consent from the patient reported in this article for publication of the information about her that appears within this Case Report and associated supplementary material.
