# Supplementary material for: Fostamatinib and the risk of acute aortic dissection in immune thrombocytopenia
Source: Br J Clin Pharmacol. 2025 Sep 17;91(12):3537–41. doi: 10.1002/bcp.70289 (PMC12648356; doi:10.1002/bcp.70289)
Supplement: Supplementary file 1 — Data S1. Histological and Immunostaining. [file BCP-91-3537-s001.docx]

**Supplemental 1: Histological and Immunostaining**

Aorta were collected in cold 1X PBS and fixed overnight in 4% PFA. Samples were then rinsed in 1X PBS and dehydrated in progressive ethanol baths until complete dehydration in 100% ethanol, equilibrated to xylene (twice, 1 hour each), embedded in paraffin and stored at 4°C. 10 μm thick sections were made using a microtome and mounted on SuperFrost Ultra Plus GOLD slides. Sections were washed in 1X PBS and fixed 10 minutes in 2% PFA. For Masson’ trichrome staining, sections were stained with Trichrome Stain Kit (Masson) (Merck #HT15-1KT). For immunostaining, sections were permeabilized with 0.1% Triton X-100 (Sigma-Aldrich) in PBS for 20 minutes, followed by incubation in an excess of blocking solution (1% bovine serum albumin, Sigma-Aldrich, A7906; 1% inactivated horse serum, GE Healthcare B15-023; 0.1% Tween-20, Sigma-Aldrich, P1379; in 1X PBS) for 1h. Sections were incubated with primary antibodies, anti-p-SYK (Cell Signaling; #2770; 1/200) and anti-αSMA (ThermoFisher Scientific; MA5-11547; 1/200) diluted in blocking solution overnight at 4°C. After rinsing in PBS, heart sections were incubated with secondary antibodies AlexaFluor (ThermoFisher Scientific; A21429 and A31571) diluted to 1/500 in blocking solution containing 300 nM DAPI (4',6-diamidino-2-phénylindole) at room temperature for 2 hours. After washes in 1X PBS, slides were mounted in Fluoromount-G (ThermoFisher Scientific, 00-4958-02). Sections were imaged using an AxioScan 7 (Zeiss) microscope device.
